# COVID-19 in Long-Term Care Facilities With Advanced Practice Registered Nurses

**DOI:** 10.1093/geroni/igab046.225

**Published:** 2021-12-17

**Authors:** Tracie Harrison

**Affiliations:** The University of Texas at Austin School of Nursing, Austin, Texas, United States

## Abstract

The purpose of our report is to give our analytical description of COVID-19 response. The study placed two APRNs in each of five participating nursing facilities (NF), 5-7 days/week for up to two years with the purpose of quantifying if employment of a full-time, salaried APRN, as part of the NF care team, improved the quality of care for the NF residents. Secondarily, we studied policies that influenced ability to provide APRN care in the NF. Resident data collected and evaluated to determine if this model of care reduced the rates of adverse events among NF residents. Data included re-hospitalizations, inappropriate prescribing (antipsychotics, antibiotics, opioids and polypharmacy), observational data, and clinical outcomes negatively affecting the quality of life for NF residents (such as falls and pressure injuries). Factors influencing APRN employment in the NF setting were NF mission, environmental aesthetics, resources, NF administrator interactions, state law, and medical director support.

